# ‘It's Skin Cancer’… a Rollercoaster of a Journey for Teenagers, Young People and Their Significant Other

**DOI:** 10.1111/jan.17070

**Published:** 2025-06-03

**Authors:** W. Mcinally, E. Hainsworth, J. Brodie, J. Nobbs, J. C. Chisholm, E. Thistlethwayte, S. Cruickshank

**Affiliations:** ^1^ The Open University Milton Keynes UK; ^2^ Applied Health Research Group The Royal Marsden NHS Foundation Trust London UK; ^3^ Skin and Melanoma The Royal Marsden NHS Foundation Trust London UK; ^4^ The Royal Marsden NHS Foundation Trust London UK; ^5^ Sutton and Institute of Cancer Research Sutton UK; ^6^ Teenage & Young Adult ANP, Ambulatory Care Children and Young Peoples Cancer Services, 3rd Floor Macmillan Cancer Centre London UK; ^7^ University of Stirling Stirling UK

**Keywords:** interpretative phenomenological analysis, melanoma, nursing, qualitative, significant other, supportive care, teenagers, young adults

## Abstract

**Aim:**

To explore the lived experience of young people aged 16–24 years diagnosed with melanoma and that of their significant other in England.

**Design:**

Interpretive phenomenological analysis.

**Methods:**

Data were collected between August 2023 and January 2024 from one specialist cancer centre in England. Thirteen young people were approached, and 10 took part. Each young person was asked to nominate a significant other. Five nominated a significant other, and five nominated no one. Although interviews were offered face‐to‐face, virtual was the preferred method. In‐depth semi‐structured interviews were audio‐recorded with the participant's consent. Interview data were transcribed verbatim and analysed.

**Findings:**

The core conceptual thread woven throughout the findings was ‘It's like being on a rollercoaster,’ which is representative of the ups and downs of the treatment trajectory, often without the support of age‐appropriate specialist care. Four superordinate themes were identified: ‘Is something wrong?’, ‘Suddenly it's serious’, ‘Out on a limb’ and ‘Finding our place’.

**Conclusion:**

Although most young people were treated in a primary treatment centre for adults with cancer, their experience was challenging from route to diagnosis through their treatment and beyond. Few received age‐appropriate care to support their physical, emotional, and social wellbeing to help them navigate the experience.

**Impact:**

There is limited evidence exploring the experiences of teenagers and young adults living with melanoma or that of their significant other. This enriched understanding supports improvement of the care pathway and service delivery for these young people and their families.

**Patient and Public Involvement:**

One young person with lived experience was paid as a consultant to be part of the research team. He helped develop the grant application and research questions, data analysis, and writing this paper.

## 1 Introduction

Approximately 2400 young people 15–24 years are diagnosed with a malignancy each year in the United Kingdom (UK) (Cancer Research UK (CRUK) 2024). Cancer is the leading cause of death from disease among this age group (O'Hara et al. 2015). Worldwide, melanoma in the adult population is a cancer that has steadily increased over the past 50 years and is increasing in young people aged 16–24 years (Miller, Wojcik, et al. 2020). Considerable evidence exists of the aetiology of the disease, optimal treatment and the impact on the prognosis of delays in diagnosis (Hajdarevic et al. 2014; Bird et al. 2015). This contrasts with the lack of evidence examining the long‐term effects of treatment for the more aggressive stages of melanoma and the low recruitment rates of teenagers and young adults with this disease receiving access to clinical trials (Miller, Fidler‐Benaoudia, et al. 2020). Whilst there is a significant amount of biomedical empirical research on melanoma, there is a dearth of qualitative research around the lived experience of teenagers and young adults and that of their significant others (McInally et al. 2021). Existing evidence suggests that teenagers and young adults and their families feel isolated, alone, and unsupported, with many treated outside a cancer specialist centre or without access to age‐appropriate care (McInally et al. 2021; Lidington et al. 2021).

The risk of developing melanoma increases with age, and it is very rarely diagnosed in children under 10 years old, accounting for only 0.7% of all cancers diagnosed in this age group (Cancer Research United Kingdom 2024; Miller, Wojcik, et al. 2020). It is the third most common cancer in teenagers and young adults 15–24 years of age within the UK (National Disease Registration Service (NDRS) 2024) and is more common in females than males. Melanoma has five main stages, ranging from stage 0 to IV. The categorisation or staging, provides a standard way of describing cancer, assisting healthcare professionals in discussing, treating, and consistently managing the disease (McInally et al. 2021).

From data collected in 2019 in England, 88 teenager and young adults had Stage I melanoma, 10 with Stage II, four with Stage III, and 15 cases not recorded. Comparable data with a breakdown per stage is unavailable in other nations (NDRS 2024). Young people appear to be diagnosed at an earlier disease stage than in previous years (Miller, Wojcik, et al. 2020). Historically, advanced melanoma was an intractable and untreatable disease, whereas today, it is revealing its molecular weaknesses, and young people with advanced melanoma are living with and beyond treatment (Zebrack 2024; Levy et al. 2018).

Discovering the mole or seeing a change in their existing mole is often how this disease presents itself. Young people undergo many forms of developmental transitions, both physiologically and psychologically, as they progress through childhood, adolescence, and adulthood. This contributes to their sense of identity, increasing independence, autonomy, and responsibility (Soanes and Gibson 2018). Given the significant ongoing changes, it is common for young people not to notice the change in their moles, but other people, such as the hairdresser, friends, or family, spot these changes.

Due to the nature of the treatment, melanoma is a cancer that does not usually require frequent inpatient stays, unlike many other forms of cancer. Consequently, young people can be treated in various settings, including outpatient departments, children and young people's services, adult non‐specialist, adult specialist centres, or teenage and young adult units (Taylor et al. 2024; McInally et al. 2021). It is now well established that young people with cancer have distinct needs in comparison to children and older adults, which are, in part, an influencing factor in this population having poorer outcomes (Lea et al. 2018).

Models of care for teenagers and young adults with cancer in the UK have developed over the last 30 years to improve survival rates and provide an environment supportive of their individual needs (Taylor et al. 2024). Specialised services for children and young people being treated for cancer within the National Health Service (NHS) have been mandated in England since 2005 by the National Institute for Health and Clinical Excellence (NICE 2025). Teenagers and young people aged 16–18 years with a cancer diagnosis must be referred to a Principal Treatment Centre for their treatment (NHS England 2023). Those aged between 19 and 24 years should be offered the choice of treatment in their nearest primary treatment centre or a local teenage and young adult –designated hospital (NICE 2025). The service specifications also require that all teenage and young adult patients with cancer be discussed in the specialist teenage and young adult multidisciplinary team meeting. Evidence from patients diagnosed from 2012 to 2014 indicated that not all patients were referred to the teenager and young adult multidisciplinary team, particularly if treated in local services (Fern et al. 2021).

Understanding the lived experience of teenagers and young adults diagnosed with this disease and at the same time trying to continue with their lives is vital to ensure the provision of care that is supportive of their physical, emotional, and social needs (Janssen et al. 2024).

## 2 Background

During the 1970s, melanoma in young people in the UK was rare, but over the intervening decades, there has been a marked increase in the reported incidence in young people around the globe (Indini et al. 2018; CRUK 2024). Among the young aged 15–24 years at diagnosis, carcinomas other than renal, hepatic, gonadal, and melanoma account for 30% of registrations from 1997 to 2016 (Public Health England 2021). Incidence is also increasing globally, and in 2019, there were 37,265 cases of melanoma in the 15‐ to 39‐year‐old age bracket (1.25/100,000) and 4248 deaths. Early diagnosis is vital for successful treatment and, if delayed, can cause treatment challenges due to the infiltration and spread of the disease (Ferlay et al. 2015).

Current evidence suggests that young people are diagnosed earlier and treated for melanoma (NDRS 2024). Empowering young people to move forward with their lives is crucial. However, limited evidence is available to support services for young people and their significant others with this specific disease (McInally et al. 2021). Despite global advances in cancer treatments, clinical management and concerted efforts to increase public awareness of skin cancer risks and prevention strategies, reported incidence continues to rise, especially within the teenager and young adult population (Hubbard et al. 2018).

For the young person, the transition from being a child to a young adult is a time of rapid and significant physical change, along with cognitive, psychological, and social development. It can be a time of emotional turmoil, rapid physical development, and growing self‐awareness (Soanes and Gibson 2018). However, this is not the only transition the young person and their significant others will experience. They will also transition to living a life after the cancer, a disease that can abruptly interrupted their life plans (McInally et al. 2021). This study aimed to explore the experiences of young people and their significant other living with melanoma. ‘Significant other’ in this study is defined as any individual highlighted as important to the young person and their cancer journey, cognisant that many may no longer be living within their parental home or may have their own family.

## 3 The Study

### 3.1 Aim

The aim was to gain an understanding and find meaning from the lived experience of each young person, 16–24 years of age at diagnosis with melanoma and that of their significant other who was experiencing this journey alongside them.

This insight is critical for nurses and other healthcare professionals to deliver specialist age‐appropriate care. Including the significant other was important to understand the additional support they find from others and gain further insight into their experiences, which may differ from that of the young person.

### 3.2 Objectives

- Describe the context in which young people receive treatment for melanoma.
- Explore the experiences of young people and their significant other during and after treatment.
- Identify each critical time point in their cancer journey.
- Work in partnership with participants to translate the findings into interventions to support young people with a diagnosis of melanoma and their significant other.

### 3.3 Design

A qualitative exploratory study using interpretive phenomenological analysis (IPA) explored the experiences of teenagers and young adults with a diagnosis of melanoma and their significant others. Interpretive Phenomenological Analysis is a branch of phenomenology seeking to capture participants' experiences and understand and identify key themes through an IPA process. It is concerned with capturing people's accounts and reflections to explore the meanings and make sense of them (Van Manen 1990). This methodological framework was developed in health psychology but has recently been used in the wider health and social science disciplines such as nursing. This approach illuminated and steered the inquiry, enabling a rich understanding of the participants' cancer trajectory.

### 3.4 Sample/Participants

Although the initial target sample size was 15 young people with any stage of the disease and 15 significant others, identifying these participants was challenging. This was the target set from the Teenage and Young Adult Cancer grant as we wanted to recruit from two NHS specialist cancer centres in England. Young people who met the study inclusion criteria (Table 1) were approached by their Teenager and Young Adult Clinical Nurse Specialist or Lead Clinical Nurse Specialist for Melanoma. Only one participant was identified in one of the specialist cancer centres, but the patient failed to follow through. At the other centre, 13 young people were approached, with 10 agreeing to participate. Seven were female (*n* = 7) and three (*n* = 3) were male.

**TABLE 1:** Inclusion criteria.

Inclusion	Exclusion
Aged 16–24 years at time of diagnosis	< 16 years and > 24 years at time of diagnosis
Up to 5 years since diagnosis	Over 5 years since diagnosis
Any gender	Young people with severe cognitive impairment
Confirmed diagnosis of malignant melanoma (newly diagnosed and/or recurrence), any stage	A diagnosis of another cancer
Ability to speak, read and write English	Unable to speak, read and write English

One had all treatment given at the local hospital, and seven had surgery in their local hospital with follow‐up at the adult primary treatment centre. Two had all treatment at the adult primary treatment centre with access to a Teenage Cancer Trust unit. All the young people approached reported that they had a diagnosis of melanoma, were Stage I to III, aged between 16 and 24 years at diagnosis, and were between 19 and 29 years of age at the time of interview (Table 2). Only five identified a significant other, three mothers and two partners (Table 3). One young person was alone, whilst four did not identify a significant other. This small sample size is considered appropriate for IPA (Smith et al. 2009).

**TABLE 2:** Characteristics of young people.

Young person	Where treated	Employment status during treatment	Age at interview	Gender	Treatment	Years since diagnosis
Jan	Primary excision at local hospital, completion & follow up at Adult PTC and TCT daycare	Studying	28	F	Wide local excision, sentinel node biopsy, adjuvant immunotherapy	5
Ella	Primary excision at local hospital and follow up at Adult PTC	Working	28	F	Wide local excision, sentinel node biopsy	4
Sue	Primary excision at local hospital, completion of treatment and follow up at Adult PTC	Not working or studying	29	F	Wide local excision	5
Jo	Primary excision at local hospital, completion of treatment and follow up at Adult PTC	Studying	25	F	Wide local excision, sentinel node biopsy	1
Evelyn	Primary excision treatment & follow up at Adult PTC	Working	28	F	Wide local excision	4
Sam	Primary excision at local hospital completion of treatment and ongoing follow up at Adult PTC	Working	23	F	Wide local excision & node clearance, treatment immunotherapy	1
Becky	Primary excision, treatment and follow up at Adult PTC	Working	28	F	Wide local excision	5
Jim	Primary excision and treatment in local hospital. follow up at Adult PTC.	Studying	29	M	Wide local excision, surgery ×2, node dissection, adjuvant targeted & immunotherapy	3
Eric	Primary excision and surgical care at local hospital, treatment and follow up TCT daycare	Studying	20	M	Wide local excision and sentinel node biopsy, adjuvant immunotherapy	4
Liam	All treatment and follow up at local hospital (discussed at PTC MDT)	Working	25	M	Wide local excision and sentinel node biopsy	4 months

**TABLE 3:** Characteristics of significant other.

Significant other ID	Gender	Role within the family unit
Shirley	Female	Mother
Brenda	Female	Mother
Carol	Female	Mother
Tim	Male	Fiancé
Brian	Male	Husband

## 4 Data Collection

Data collection commenced from August 2023 to January 2024. Interviews were offered via the Microsoft Teams platform, telephone, or face‐to‐face, depending on the participant's preference, and recorded digitally. All participants chose Microsoft Teams (*n* = 12) or telephone (*n* = 3). The interviews took approximately 60 min. An experienced qualitative researcher (E.H.) not working in cancer care conducted the interview guided by an interview schedule. Table 4 provides a sample of the types of questions asked to all participants. Travel expenses were for stakeholder events and an honorarium of £25 Amazon vouchers per participant.

**TABLE 4:** Topic guide for interview questions.

To begin with, I would like to learn a bit about your journey to being diagnosed with malignant melanoma. What things stick in your mind about the time leading up to diagnosis? What stands out for you from your experience through this journey from diagnosis to treatment or after treatment? (Probes: diagnosis, treatment, treatment setting, after treatment) How has this cancer affected your life? (Probes: Self, daily life, work, social, relationships …) How has your life changed as a result of your cancer? (Probes: negative and positive changes?) What are your main concerns at this time? How do you feel about the support you have at the moment? What support do you need just now? (Probes: family, nurse specialist etc.?) Is there anything else you would like to talk about in relation to your experience of living with malignant melanoma that we have not covered?

## 5 Ethical Considerations

An NHS Foundation Trust Clinical Research Committee (CCR5759) reviewed this study. The NHS was the sponsor. This study received a favourable ethical opinion from South‐East Scotland RECO1 (23/SS/0048) and approval from NHS Health Research Authority. All participants were given a pseudonym to maintain anonymity. Participants were assured that the information they provided would be confidential and that they had the right to refuse to participate in the study at any time. A debriefing letter was provided with details of supportive resources being provided after the interview.

## 6 Rigour

Guba and Lincoln (1994) identified four measures of rigour to assess qualitative methodologies and ensure their trustworthiness. These measures of credibility, transferability, dependability and confirmability were applied to the research. To ensure the credibility and transferability of the findings, an expert in the field delivered a workshop on IPA, which helped with the data analyses and the interpretation of the narrative into sub‐themes and themes. The dependability and confirmability of the data analysis were evaluated by the IPA expert who is part of the team (W.M.).

The researchers strove to be reflexive throughout the study and be aware of any personal bias and experiences, mindful of their experience as young people with melanoma and cancer specialists. A research forum was set up where the research team met regularly virtually and face‐to‐face to reflect and support the IPA element of the study. Within IPA, bracketing is recognised as not being entirely possible or desirable (Smith et al. 2009). Being transparent and reflective throughout the research process supported the participant's narrative and our understanding to ensure that the interpretations of the participant's stories were grounded and authentic. It was important to stress what the dialogue brings to the text and what the text brings to the research (Smith et al. 2009).

A stakeholder event was a key element of the preliminary validation and dissemination of the findings held in October 2023, where all the participants, Clinical Nurse Specialists, Nurse Consultants, and key stakeholders for Teenage and Young Adults with Cancer and Teenage Cancer Trust participated. This event was a participatory workshop where the research team presented the findings through moderated and facilitated discussions. The main objective was to bring the participants' narratives and conversations into a safe space, check the research team's interpretation, and engage with a broader audience that influences the care of teenagers and young adults with cancer.

## 7 Data Analysis

Following the IPA methodology, interviews were transcribed verbatim and analysed using a systematic inductive interpretative approach, followed by the recognised six‐key‐step approach (Table 5) (Smith et al. 2009). Two researchers (W.M. and E.H.) were responsible for the iterative process. Throughout the iterative analysis process, we strived to explore the essence of what the participants felt was real, true, and important to them at this time. This reflected IPA's epistemological and ontological foundation (Smith et al. 2009). In understanding that the experience is subjective, and that IPA is concerned with the meaning of what people experience as a phenomenon rather than a direct reality, this was addressed through continuous adherence to the hermeneutic and idiographic nature of the IPA approach. Data analysis rigorously drew upon the three philosophical perspectives: phenomenology (examining the lived experience), hermeneutics (interpretation of the lived experience) and idiographic (attention to particulars of individual stories). In the findings, the narrative representing the whole group must always be traced back to the individual level. Larkin et al. (2019) posit that there must always be a balancing act between description and interpretation when conducting an IPA study.

**TABLE 5:**
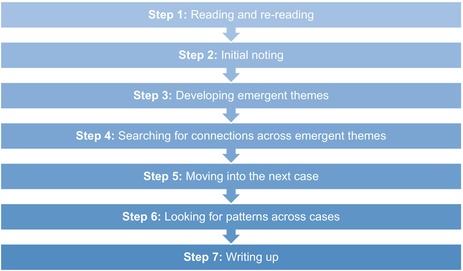
Key steps to data analysis adapted by Smith et al. (2009).

Each transcript was analysed separately for the young people and the significant other. Analysing the data individually allowed the narrative to ‘open up’ and reveal the participants' experiences as various ‘individual parts’ and then as a ‘whole’ (Smith et al. 2009). One of the team (W.M.) is also a member of the National IPA group, and this was supportive in ensuring the methodology was followed.

Working within the hermeneutic circle, constantly and dynamically moving between the individual experiences along with the data set as a whole, we carefully selected individual comments and narratives from specific participants to illuminate each of the sub‐and superordinate themes while being mindful of the idiographic nature of the study. It was clear that each young person and their significant other had established dynamic interconnections built around pre‐existing inter‐relationships and therefore the data was presented together.

Steps 1–3, shown in Table 6, illustrate how each sentence and section of the transcription was subsequently examined to expose meaning and identify the experiences of each participant. Utilising software tools, individual interviews were analysed and colour coded. Themes from the individual transcripts were identified so that the idiographic nature of IPA is maintained. The themes were then grouped into similar themes, as seen in Step, Table 6 to develop the superordinate themes. The two researchers (E.H. and W.M.) discussed the findings with the research team to ensure rigour and credibility (Smith et al. 2009; Vicary and Ferguson 2024; Yardley 2000).

**TABLE 6:** Steps 1 and 2—reading, re‐reading, and initial noting.

Transcript: Shirley	Participant	Comments	Themes emerging
Can you tell me how this news was given to you?	Researcher		
**The way we found out was that when we got the results, it was never a good sign, because there was like a carer person with the doctor. There was an extra person, with the doctor, you know, I guess who works with children. So that wasn't a good sign when we saw that. And I think there were about four other nurses in this tiny little room, this tiny little room with like two seats for us both and we instantly knew it was bad news**.	Jo	The way we found out Too many people in the small room Knew something was wrong Worried and anxious The environment sticking in head	Trusting others How the news was communicated How healthcare professionals were acting

Step 3: Developing emergent themes					
Transcript: Jo			Participant transcript: Sam		
Yeah, it was bad actually. So, I'm from a really medical family, so I've always really trusted GPs and doctors and kind of whatever they've said—I've gone with—and for some reason I had this mole on my arm that was just making very anxious, and I went to my GP, and he said stop being silly. It's nothing. I went back to my GP, he said it's nothing and I ended up going, I think it was four times			Then 2 months later I did the same thing again, I went to another dentist, and by this time it had doubled in size again and it was feeling like a little bit tender. And this dentist said Oh, I bet there's some food stuck in your mouth, so I'm going to cut it open, and with no like numbing or anaesthetic, just took sort of like a sharp tool and started having a go at it		
Example			Example		
Interpretation review:			Interpretation review:		
Description/Content:	Language use:	Interrogative coding:	Description/Content:	Language use:	Interrogative coding:
Do you know what it was that made you feel so troubled by the mole?	It was rock solid and I've got quite a lot of moles and freckles. I'm very freckly But this was the colour of my skin and it kind of developed relatively recently	Change in moles quite freckly. Sensing something was wrong. Change in skin. Noticing the change	We were talking about how you had gone about getting the mole removed.	I remember the surgeon who removed the mole, she said, oh normally I receive the results in a couple of days, I'm sure everything's fine	Nothing really to worry about, but Surgeon also puzzled by not having results. Ambiguity

Definition of terms used:	
Description/content:	What the interviewer was asking from the participant
Language use:	Data from the original transcript
Interrogative coding:	Asking questions of the data to facilitate a deeper coding and inquisitive attitude towards the data

Step 4—searching for patterns across	
Colour coding	
Blue	Journey to suspected mole/what is this? Back and forth
Orange	Anxious and worried, trying to cope
Burgundy	Appearance of the mole
Red	Unsure/uncertainty/what the ……!
Violet	Supportive surroundings/family/friends/partner/supportive networks/trust
Purple	Bad news being given
Yellow	Bewildered/surprised/grappling with reality/loss, fear
Brown	Changes to self
Teal	Unusual for age group
Dark blue	Treatment experience
Pink	Financial hardship/being a student
Light Blue	The future/something positive/using the experience to shape the present/future
Sky blue	Expectations/the ‘norm’/finding out what ‘it’ is?
Dark green	Being young/can't be you are young!
Mustard	Disruptions to my life, paying the price impact not taken seriously
Black	Familial/genetic
Blue/Grey	Being aware and having knowledge
Turquoise Green	Recurrences, not just the one cancer, more lumps
Pale pink	Scarring, disfigurement of body
Brown	Health awareness, increasing paranoid about any sign of ill health or potential skin change
Dark purple	Looking ‘normal’ ‘not a real cancer’
Light pink	Exposure to the sun/tanning beds/climate/ignorance
Lime green	Communication/no one listening
Peach	Being a carer felt alone

## 8 Findings

**FIGURE 1:**
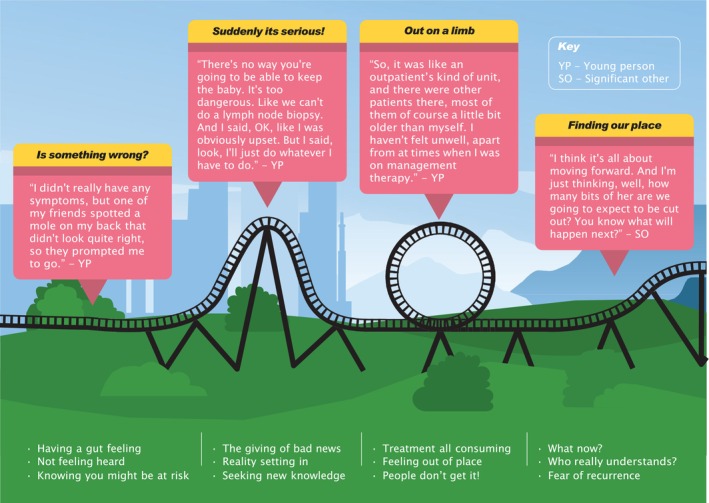
The core conceptual thread—“It's like being on a rollercoaster”.

Four superordinate themes were identified comprising of 12 sub‐themes: (1) ‘Is something wrong’ capturing the feeling that something wasn't right, (2) ‘Suddenly it's serious’ represented a realisation that their diagnosis was potentially serious, (3) ‘Out on a limb’ expressed their feelings of isolation and being different to other patients with cancer (4) ‘Finding our place’ encapsulated feelings of looking forward to the future but with fear of recurrence. The core conceptual thread woven throughout the findings was “It's like being on a rollercoaster,” highlighting the superordinate themes and sub‐themes. It represents the cancer trajectory, the ups and downs of a cancer diagnosis, and the treatment experience. Figure 1 illustrates the journey, with its highs, lows and changing momentum, reflecting the wide range of experiences. For all except one young participant and their mother (*n* = 2), the roller coaster would go around again when more unwelcome news is shared.

### 8.1 ‘Is Something Wrong’

This theme reflects the beginning of their journey from discovering the mole to seeing a change in their existing mole(s) through their daily routines. This theme captures participants' initial sense and gut feeling that something was not right. Both the young people and significant others described the appearance of the skin lesion on various parts of their body, not always where the ultraviolet light would affect the skin, as ‘quick growing’, ‘itchy’ or ‘sore’ and this caused them anxiety about the need to get it seen to by a healthcare professional. Not all the participants identified that it was a mole on their skin that was changing until it became darker and began to itch and bleed. As one young person and a mother expressed:

It just appeared out of nowhere like it appeared quite quickly (Jo)

And on one of those kind of checkup appointments she had, mentioned this mole. I never saw it. It was tiny and quite quick growing (Brenda).

Some described repeated presentations to the General Practitioner or Dentist, which led to feelings of frustration. The delays were further compounded by not being taken seriously and General Practitioner not hearing their concerns.

I went to my GP, and he said stop being silly. It's nothing. I ended up going back, I think it was four times (Sue).

These experiences emphasise the need for greater knowledge and awareness about skin cancer for teenagers and young adults and healthcare professionals and acting on removing suspicious moles to avoid delays in diagnosis.

### 8.2 ‘Suddenly It's serious’

At the point of receiving the news about their diagnosis, most participants described a change in the nature and tone of consultation, which indicated to them that this situation had suddenly become serious. An example of this includes how additional staff members were present in the room, and the young person described being asked if they had someone to join them for the appointment. As participants recalled:

And I think there were about four other nurses in this tiny little room, this tiny little room with like two seats, and we instantly knew it was bad news (Sam)

I didn't expect it. I don't think either of us did really.

Because again, our knowledge of this particular type of cancer was not very big at all (Shirley)

For most of the participants, the severity of the situation was not communicated, and healthcare professionals used medical terms that were unfamiliar to them. One young participant left the consultation after his biopsy, not knowing that melanoma was, in fact, skin cancer, as he explained.

He did not mention cancer at all once, so the next few days I went around school telling everyone it was ‘just melanoma’… I was at my friend's house, and I was talking about it, and then his mum overheard, and then she's like, you mean cancer. And I was like, no, no, I don't think so. I don't think it's cancer (Jim).

Information received regarding the treatment was alarming for some of the participants, bringing the situation's unexpected seriousness for a significant other whose partner was expecting a baby and recently found out that they had melanoma.

It sort of raised alarm bells, as they were telling us we might not be able to keep the baby (Tim).

Participants commented on the contrast between their sense of normalcy on the way to their consultation and the change of gears to receive crucial information about their skin cancer. At the point of diagnosis, clinical interactions and interventions picked up pace. Many went on to seek knowledge in the form of blogs and social media to try and find out more about the experience of melanoma for young people from first‐person accounts rather than from statistics. The young female participants and their partners became anxious waiting for information about treatment decisions, which seemed to take too long. They described waiting for the regular multidisciplinary meeting before results are shared or decisions communicated to them. They expressed some uncertainty and mistrust about what they had been told and perceived a lack of accessibility to the healthcare team to find things out. The period of waiting for news was a painful time of pause and limbo, a loop of anxiety on their journey.

### 8.3 ‘Out on a Limb’

Following on from ‘suddenly it's serious’ was this sense of isolation and being different. Young people felt they were not ill, unlike other patients with cancer, but they had started to realise the seriousness of the disease and the type of cancer they had.

You know, a 20‐year‐old to be walking in there and kind of be surrounded by … people who look very sick and very vulnerable when you're not feeling sick or vulnerable. Yeah, it makes you feel a bit like an imposter (Ella).

Everyone just automatically assumed that because I had cancer, I'd have to have chemotherapy, and everyone is talking to me about losing my hair and stuff (Evelyn).

Participants described melanoma as a cancer that other people around them did not properly understand, and this could make them feel isolated and often alone.

I think even with my family and close friends and stuff, I think like people don't get it. I think there's a misconception that it's only the bit on your skin which has got it. It's like any cancer. Once it's inside your body, it spreads, or it's got the potential to spread (Sue).

The treatment is different for this type of cancer, and many of the young people, after surgery, spend time waiting to be seen at their follow‐up appointment.

Yeah, I couldn't swallow. I couldn't drink. I couldn't eat for about two months, and it was very painful but apart from the regular check up there and visit to GP there was nothing (Liam).

Young people also described how this interruption had placed heavy emotional strain on their immediate family and friends. There was this sense of helplessness and frustration that they were unable to support.

He was studying abroad, and we were back home, so it was hard for him and us (Carol).

### 8.4 ‘Finding Our Place’

This final theme illustrates how the participants adjusted their lives in several ways. It had changed, but for most, they had more awareness about cancer, especially skin cancer. However, there was also the fear of recurrence through trying to find their place back in society, most described a positive outlook in life with a new perspective and a greater appreciation of the present moment. Others described a new and ongoing heightened anxiety and paranoia about their health and the possible recurrence of their condition. This could pick up pace and become particularly acute when regular follow‐up ends. For one young participant who was not responding to treatment, their overriding concern was whether their disease could be curable.

I think that this might not be curable—is the main thing I worry about, and I'd say that pain has changed my life, like post‐surgeries and things like that. I'm in a lot of pain and I can't do anything for myself. I can't (Sam)

However, others were trying to be upbeat and to make the best of life moving forward for their child or partner, as one mother explains about her daughter starting her career.

She's just done exams. Everything came together. I just hope that she's passed all her exams. You know, it was a difficult stage. She works hard and sometimes she says you know I've had enough but the next day she says I love my job. You know she's a 25‐year‐old it's normal (Shirley).

Finally, those whose lives had returned to a form of normality encountered problems and conflicting advice relating to concerns about contraception, travel insurance, tattoos and laser hair removal. Because of their prior melanoma diagnosis, insurance companies and external organisations treated them differently, which struck them as an unwelcome and persistent reminder of the impact of their cancer on their lives.

And I've been told that I couldn't get travel insurance. Nowhere will give you like laser hair removal treatment if you've had skin cancer—so all of these things, like, I keep coming across in life that just feel like a horrible shadow of like what happened (Ella).

The ongoing impact of melanoma on their lives was also identified as a factor for significant others. Parents and partners talked about their fear of potentially more wide‐reaching impacts and future difficulties such as recurrence, fertility, and the wider implications for other family members (for those with genetic mutations), as one mother explained.

Now we must be extremely careful with the sun. But her sister doesn't understand the total implication of being in the sun and ‘You start thinking about, you know will things be very complicated for her?’ And I'm just thinking, well, how many bits of her are we going to expect to be cut out? (Carol).

## 9 Discussion

This study identified key concepts relevant to young people and their significant other living with melanoma, providing narrative insights into the journey with this disease. Although young people's and significant others' experiences were interconnected, some experiences were unique to the significant other. The unique experiences related to the tension that mothers experienced in consciously stepping back and allowing young people to make their own decisions, whilst feeling that they could anticipate bigger picture issues that their child could not. The partners described the frustration and powerlessness of waiting for healthcare updates often after the news had been relayed to the young person. Although unique to the significant others, these experiences could be encapsulated with the young person's anxiety and stress.

Whilst elements of this rollercoaster are not dissimilar to the experiences of all teenagers and young adults with other cancers, there are distinctions to having a melanoma diagnosis. Teenager and young adult cancer care has evolved to become a speciality, with specialist age‐appropriate units. There are 28 teenage and young adult units across the UK with healthcare professionals educated and trained in this area of care (Smith et al. 2016). Clinicians within the teenager and young adult multidisciplinary teams recognise the unique needs of this patient group as they navigate the challenges of adolescent and young adult development whilst contending with a cancer diagnosis (Soanes and Gibson 2018; McInally et al. 2021). The needs of young people are reflected in NHS England's service specifications, which specify the provision of teenage and young adult Cancer Care (NHS England 2023). However, a young person with melanoma will typically attend their General Practitioner or an adult outpatient setting where a biopsy or excision of the skin lesion takes place, which yields the diagnosis of melanoma (Teenage Cancer Trust 2025). The young person may go on to further surgery in their local adult service or be referred to the primary treatment centre for ongoing care. Therefore, the road to diagnosis for the participants within this study was often stressful, typified by the provision of information from healthcare professionals who were not expert in teenager and young adult melanoma care. In addition, many faced delays in diagnosis, exacerbated by numerous appointments and repeat presentations to healthcare services before the situation was taken seriously.

Following diagnosis, most participants had heightened awareness about their health and future risks as the rollercoaster accelerated from ‘Is something wrong’ to ‘Suddenly it's serious.’ Their experience then changed course to ‘Out on a limb’ where the participants expressed feeling out of place and having a sense of feeling and looking different. The rollercoaster still rises more slowly after treatment to capture ‘Finding our place’, demonstrating an uncertainty of life in general but still looking forward (Al Omari et al. 2017; Soanes and Gibson 2018).

There were differences in the experiences of those who had surgery alone and those who also had other treatment, which impacted the level of support received. Those receiving surgery alone found their experience was short but intense, with a sense of abandonment afterwards. This is typical of many young people with melanoma who may have little or no contact with hospital services following surgery (McInally et al. 2021; Boutros et al. 2024). The physical treatment from healthcare professionals often takes precedence over the emotional and social aspects, leaving young people feeling alone and frightened long after treatment finishes and supportive services are less available (Davies et al. 2018; Grinyer 2007, 2009).

The current documented survival rate for teenager and young adults diagnosed with melanoma is 95% if diagnosed early, allowing effective treatments and interventions to be introduced, improving outcomes (Cancer Research United Kingdom 2024; Refolo et al. 2025). There has been a seismic shift in the treatment for melanoma, i.e., immunotherapy and survival outcomes have improved considerably (Knight et al. 2023); however, uncertainty remains over how best to support teenagers and young adults as they get older or indeed live with side effects from treatment (Zebrack 2024; Levy et al. 2018). This population has distinct needs regarding fertility, working patterns, finances, fear of recurrence with ‘patchy’ follow‐ups with health professionals, and depending on the stage of disease and treatment pathway (Janssen et al. 2024). This study has identified many of these needs, however additional research is needed to gain further insight into this area of care.

There is increasing recognition in the UK that healthcare should be tailored to meet the needs of young people and their families (Lea et al. 2018; Taylor et al. 2020). However, this was not evident among the participants interviewed in this study. Considerable investment has been made into setting up teenage and young adult units across the UK (Smith et al. 2016; Taylor et al. 2024) and to ensure that healthcare professionals caring for teenagers and young adults have the knowledge and skill set to care for them (Smith et al. 2016; Lea et al. 2018). In 2010, about two‐thirds of those aged 15–18 years and one‐third of 19‐ to 24‐year‐olds were believed to have had contact with a teenage and young adult primary treatment centre (O'Hara et al. 2015). It was unclear why so few of them were aware of these specialist services despite such investment and publication of the NICE (2025) guidance.

Young people's experience of cancer often occurs at a time when they are in the process of developing their early adult life plans and are in the process of developing their adult selves, gaining greater independence, and making life plans (Cable et al. 2023; Davies et al. 2018; Weston et al. 2018). Davies et al. (2015) advocate empowering young people to make decisions but negotiate with the family/significant other where possible. Nine of the 10 young people interviewed lived independently and were empowered to care for themselves.

Traditionally, research in teenagers and young adult cancers has described them as one homogenous group. Treatment is primarily inpatient for some tumour groups, such as haematological or solid tumour malignancies. As discussed previously, young people with low‐stage melanoma are usually seen by the General Practitioner or outpatients under dermatology and therefore their needs may be overlooked (NICE 2025; McInally et al. 2021). The evidence suggests that only a few young people referred to dermatology services with suspicious moles have melanoma (Refolo et al. 2025). This highlights the importance of referral to the teenager and young adult multidisciplinary team for consideration of their holistic needs. This study was able to pick up the nuance of teenagers and young adults and their specific experiences, which may differ from other teenager and young adult cancer groups. For example, a young person with leukaemia will receive regular aggressive adjuvant therapy and often is in hospital for most of their treatment with side effects, such as hair loss, low haemoglobin and platelet counts. By contrast, young participants with melanoma felt out of place in wards, where they did not look like a patient with cancer. Although over the last decade, through the BRIGHTLIGHT study (Taylor et al. 2020, 2024), evidence suggests that the benefits of specialist age‐appropriate care in England are more nuanced than merely the provision of care within teenager and young adult specific units, with the outcomes associated with such specialist care remaining under evaluation. In 2010, about two‐thirds of those aged 15–18 years and one‐third of 19–24 years were believed to have had contact with a teenage and young adult primary treatment centre (O'Hara et al. 2013). In the current study, only two young participants and their significant other reported contact with teenager and young adult specialist services to access support in navigating their cancer journey and beyond.

## 10 Strengths and Limitations

This study focused on one cancer and provided rich and detailed data of young people and their significant others. The researchers have provided a valuable perspective through the carefully chosen individual narrative providing insights from teenagers and young adults and their significant others with melanoma. The study has several limitations. First, the findings of this study are from 10 young people and only five significant others from one hospital in England. This is in stark contrast to an earlier study by McInally et al. (2021), whereby all participants identified a family member. It might be due to the older age group included in this study and/or personal choice. Secondly, we found this population, aged 16–24, difficult to identify through the clinical nurse specialist or hospital services. Thirdly, there were only two young people who had access to age‐appropriate specialist care after diagnosis. Lastly, participants were interviewed once, and the experience was only captured at one specific point in the pathway.

## 11 Conclusions

All teenagers and young adults with cancer should be discussed in the regional teenage and young adult multidisciplinary team to ensure their needs are met. However, not every patient needs to be seen at the primary treatment centre, and patients can be linked to the teenager and young adult clinical nurse specialist and the healthcare team. Therefore, the care delivery for teenagers and young adults and their significant others requires stronger links and communication channels between services, at the beginning, during, and after treatment to address these challenging and isolating experiences. The core conceptual thread can help healthcare professionals better understand the multifaceted and dynamic physical, emotional, and social needs of young people and their significant others in living with melanoma.

As survival outcomes increase and the use of immunotherapy and targeted agents in the treatment of advanced melanoma becomes standard practice, it is evident that further multi‐centred research is needed to gain further insight into the experiences of teenagers and young adults with melanoma and that of their significant other. In addition, evidence is required to know where these young people are being treated.

## Author Contributions

W.M., E.H., S.C., J.B. have made substantial contributions to conception and design, acquisition of data, or analysis and interpretation of data. W.M., E.H., S.C., J.B., J.N., E.T., J.C.C. involved in drafting the article or revising it critically for important intellectual content. All authors must have agreed on the final version of the paper and must meet at least one of the following criteria (based on those recommended by the ICMJE).

## Conflicts of Interest

The authors declare no conflicts of interest.

## Data Availability

The data that support the findings of this study are available from the corresponding author upon reasonable request.
